# Conserved roles for Polycomb Repressive Complex 2 in the regulation of lateral organ development in Aquilegia x coerulea ‘Origami’

**DOI:** 10.1186/1471-2229-13-185

**Published:** 2013-11-20

**Authors:** Emily J Gleason, Elena M Kramer

**Affiliations:** 1 Department of Organismic and Evolutionary Biology, Harvard University, 16 Divinity Ave., Cambridge, MA 02138, USA; 2 Department of Molecular and Cellular Biology, Harvard University, 16 Divinity Ave., Cambridge, MA 02138, USA

**Keywords:** Polycomb repressive complex 2 (PRC2), Compound leaves, *AGAMOUS*, Class I KNOX genes, Carotenoid biosynthesis, Epigenetics, Evolution, *Aquilegia*

## Abstract

**Background:**

Epigenetic regulation is necessary for maintaining gene expression patterns in multicellular organisms. The Polycomb Group (PcG) proteins form several complexes with important and deeply conserved epigenetic functions in both the plant and animal kingdoms. One such complex, the Polycomb Repressive Complex 2 (PRC2), is critical to many developmental processes in plants including the regulation of major developmental transitions. In addition, PRC2 restricts the expression domain of various transcription factor families in Arabidopsis, including the class I KNOX genes and several of the ABCE class MADS box genes. While the functions of these transcription factors are known to be deeply conserved, whether or not their regulation by PRC2 is similarly conserved remains an open question.

**Results:**

Here we use virus-induced gene silencing (VIGS) to characterize the function of the PRC2 complex in lateral organ development of *Aquilegia x coerulea* ‘Origami’, a member of the lower eudicot order Ranunculales. Leaves with PRC2 down-regulation displayed a range of phenotypes including ruffled or curled laminae, additional lobing, and an increased frequency of higher order branching. Sepals and petals were also affected, being narrowed, distorted, or, in the case of the sepals, exhibiting partial homeotic transformation. Many of the petal limbs also had a particularly intense yellow coloration due to an accumulation of carotenoid pigments. We show that the *A. x coerulea* floral MADS box genes *AGAMOUS1* (*AqAG1*), *APETALA3-3* (*AqAP3-3*) and *SEPALLATA3* (*AqSEP3*) are up-regulated in many tissues, while expression of the class I KNOX genes and several candidate genes involved in carotenoid production or degradation are largely unaffected.

**Conclusions:**

PRC2 targeting of several floral MADS box genes may be conserved in dicots, but other known targets do not appear to be. In the case of the type I KNOX genes, this may reflect a regulatory shift associated with the evolution of compound leaves.

## Background

Maintenance of proper gene expression in differentiated cells is essential for the development of multicellular organisms and epigenetic regulation is an important player in this process Reviewed in: [1-3]. One family of proteins with deeply conserved functions in epigenetic regulation is the Polycomb Group (PcG). The PcG was first discovered in *Drosophila melanogaster* as repressors of the HOX genes [4]. Several PcG complexes exist in both plants and animals, each with distinct functions in epigenetic silencing Reviewed in: [5,6]. However, only the Polycomb Repressive Complex 2 (PRC2) has been well characterized in multiple plant models Reviewed in: [5,7]. The main function of the PRC2 complex is trimethylation of lysine 27 of histone H3 (H3K27), a histone modification known to suppress gene expression [8]. The PRC2 contains four core proteins; the histone methyltransferase *Enhancer of Zeste* (*E(z)),* and three other proteins thought to enhance PRC2 binding to nucleosome [9]. These include *Suppressor of Zeste 12 (Su(z)12)* and *Extra Sex Combs (ESC),* known respectively as *EMBRYONIC FLOWER 2* (*EMF2*) and *FERTILIZATION INDEPENDENT ENDOSPERM* (*FIE*) in plants, and *Multi-Copy Suppressor of IRA 1 (MSI1*) Reviewed in: [10]. The *E(z)* lineage in plants has experienced an ancient duplication such that most angiosperms have at least two paralogs, known as *CURLY LEAF* (*CLF*) and *SWINGER* (*SWN*) [11]. Many plant species have additional duplications in the core PRC2 loci that allow them to form several PRC2 complexes often with distinct developmental functions [12,13].

PRC2 is involved in a number of important developmental transitions. In the plant model system *A. thaliana*, these functions include endosperm development, early repression of flowering to allow proper vegetative development, the eventual transition to flowering, and flower organogenesis [14-17]. In grasses, the PRC2 complex plays roles in floral induction (rice and barley), flower development (rice), suppressing cell divisions in the unfertilized ovule (rice), and endosperm development (rice and maize) [12,18,19]. In the moss model *Physcomitrella patens*, PRC2-dependent remodeling appear to be required for the switch from gametophyte to sporophyte development [20,21].

In addition to its role in developmental transitions, PRC2 has been suggested to function in lateral organ development in *A. thaliana*. In fact, the first description of a plant PRC2 function was discovered with the characterization of the *clf* mutant in *A. thaliana*[17]. The *clf* plants had severely curled leaves, smaller narrower sepals and petals, and partial homeotic transformations of sepals and petals towards carpel and stamen identity, respectively. Two MADS box genes, the C class member *AGAMOUS* (*AG)* and the B class representative *APETALA3* (*AP3*) were shown to be over-expressed in *clf* mutants, suggesting that the PRC2 complex was required for stable repression of these genes [17]. This was particularly interesting because MADS box genes regulate homeotic floral organ identity in plants somewhat analogously to the way HOX genes regulate segment identity in animals [22-25]. Further studies have subsequently shown that the E class MADS *SEPALLATA3* (*SEP3*) is similarly up-regulated in *clf* mutants [26]. PRC2 has also been shown to regulate the expression of the class I KNOX genes during vegetative development. The class I KNOX genes are a family of homeobox domain-containing loci in plants that have conserved roles in promoting pluripotency in the shoot apical meristem and in compound leaf development [27,28]. Katz et al [29] found that in addition to the phenotypes reported in *clf* mutant plants, *FIE* cosuppressed plants also had loss of apical dominance and fasciated stems, rolled leaves with varying degrees of serration, loss of phyllotaxy in the inflorescence, and many problems with ovary and ovule development. They further demonstrated that several class I KNOX genes, including *BREVIPEDICELLUS* (*BP*), *KNOTTED-LIKE FROM ARABIDOPSIS THALIANA 2* (*KNAT2*), and *SHOOTMERISTEMLESS* (*STM*), were over-expressed in rosette leaves of *FIE* silenced plants. In *clf* mutants, *STM* and *KNAT2* were over-expressed but *BP* was not, possibly because the *CLF* paralog *SWN* was acting redundantly. The class I KNOX genes *MOSS KNOTTED1-LIKE 2* and *5* (*MKN2* and *MKN5*) were also shown to be over-expressed in *PpFIE* mutant gametophytes [21,30], suggesting that PRC2 targeting of the class I KNOX genes may be deeply conserved.

While the functions of the floral ABC class and type I KNOX genes are thought to be conserved across angiosperms, comparative studies of their regulation have largely focused on upstream transcription factors, such as *LEAFY* or ARP family members [31,32]. In order to begin addressing the question of whether PRC2-targeting interactions are similarly conserved, we have examined the functions of PRC2 members in lateral organ development of the emerging model system *Aquilegia*. The genus *Aquilegia* is a member of an early diverging lineage of the eudicotyledonous flowering plants, the Ranunculales, that arose before the radiation of the core eudicots Reviewed in: [33]. It therefore can be used as a rough phylogenetic midpoint between *A. thaliana* and model systems in the grasses [34]. Additionally, many ecological, evolutionary and genetic studies have been conducted in *Aquilegia* over the past 50 years. These have taken advantage of its small genome (n = 7, approximately 300 Mbp) as well as a number of more recent genomic tools, including the fully sequenced *Aquilegia x coerulea* genome ( http://www.phytozome.net/search.php?method = Org_Acoerulea) Reviewed in: [33,35]. The reverse genetic tool virus-induced gene silencing (VIGS) has been optimized in several species of *Aquilegia*[36] for both leaf and floral development [37-40]. Previously we examined the evolution and expression of the PRC2 family in *Aquilegia*[41] and found that the genome contains a simple complement of PRC2 homologs: one copy each of the two plant *E(z)* homologs, *AqCLF* and *AqSWN*; an *ESC* homolog, *AqFIE*; a *Su(z)12* homolog, *AqEMF2*; and a copy of *MSI1, AqMSI1*. We initially assessed gene expression throughout *Aquilegia vulgaris* development due to its strong vernalization dependency and found no obvious tissue or stage specialization. Furthermore, the ancient paralogs, *AqCLF* and *AqSWN,* are not imprinted in *Aquilegia* endosperm as is seen in other plant species [19,41].

In the current study we have used VIGS to knock down the expression of *AqFIE* [Genbank: JN944599] and *AqEMF2* [Genbank: JN944598] in unvernalized and vernalized *Aquilegia coerulea* ‘Origami’ plants using the *ANTHOCYANIN SYNTHASE* (*AqANS*) as a marker gene. Due to limitations of the VIGS approach, it is not possible to assess many life cycle transitions, most notably flowering time, but lateral organ development can still serve as a useful model for PRC2 function. We find that PRC2 plays a role in leaf and floral organ development in *A. x coerulea,* particularly via down-regulation of the floral MADS box genes. This has allowed us to identify PRC2 targets that appear to be conserved between Arabidopsis and *Aquilegia* as well as some novel PRC2-regulated pathways.

## Methods

### Virus-induced gene silencing

The *Aquilegia* VIGS protocol was preformed as described previously [36]. TRV2-*AqCLF-AqANS,* TRV2-*AqSWN-AqANS,* TRV2-*AqFIE*-*AqANS* and TRV2-*AqEMF2-AqANS* constructs were prepared by PCR amplifying approximately 300 bp regions of each gene using primers that added *Eco*R1 and XbaI restriction sites to the 5′ and 3′ ends of the PRC products (see Additional file 1). The PCR products were then purified and cloned into the TRV2-*AqANS* construct [36] and electroporated into *Agrobacterium* strain GV101. *A. x coerulea* seedlings were grown to approximately the 4 to 6 leaf stage and then either treated as described in Gould and Kramer [36] for unvernalized samples or as described in Sharma and Kramer [37] for plants that had been vernalized for approximately 4 weeks at 4°C [36,37]. The TRV2-*AqANS* and TRV2-*AqFIE-AqANS* constructs were each used to treat approximately 400 plants over 4 rounds of VIGS. Approximately 250 of these plants were VIGS treated before vernalization and approximately 150 of these plants were treated after vernalization. The TRV2-*AqEMF2-AqANS* construct was used to treat approximately 100 plants; roughly 50 of these plants were treated before vernalization and 50 were treated after vernalization. Leaves, petals, and sepals showing *AqANS* silencing were photographed, collected, and stored at -80°C for RNA analysis.

### RT-PCR

RNA was extracted from control (*AqANS* silenced) and experimental (*AqFIE* and *AqEMF2* VIGS-treated) tissue. One half of the *AqANS* silenced (control) leaves were from separate unvernalized plants (C1 and C2) and half were from separate vernalized plants (C3 and C4). Five of the TRV2-*AqFIE-AqANS* treated leaves were from separate unvernalized plants (F2, F4, F5, F7, and F8) while three were collected from separate vernalized plants (F1, F3, and F6). All of the TRV2-*AqEMF2-AqANS* leaves were collected from separate vernalized plants (E1-E4). Sample numbers do not indicate order of leaf appearance but were collected at roughly the same stages of development. We selected a variety of observed phenotypes for each set of samples. For leaves, the RNeasy Mini Kit (Qiagen, Valencia, CA) was used. For petals and sepals RNA was extracted using the Pure-Link Plant RNA Reagent small scale RNA isolation protocol (Ambion, Austin, TX). RNA was treated with Turbo DNase (Ambion, Austin, TX) and cDNA was synthesized from 1 μg of total RNA using Superscript II reverse transcriptase (Invitrogen, Carlsbad, CA) and oligo (dT) primers. cDNA was diluted 1:5 prior to use.

Amplification was performed using AccuStart PCR SuperMix (Quanta Biosciences Inc, Gaithersburg, MD). The amplification program began with 1 minute activation step at 94°C, followed by a 20 second denaturing step at 94°C, a 15 second annealing step at 55°C, and a 15 second extension at 72°C, repeated for 30 cycles. This cycle number was chosen for optimal detection of *AqFIE* and *AqEMF2*, which are expressed at relatively low levels in mature organs, especially compared to the high expression levels of *AqIPP2*. All primers used are listed in Additional file 1. Amplification of *ISOPENTYL PYROPHOSPHATE:DIMETHYLALLYL PYROPHOSPHATE ISOMERASE2 (AqIPP2)* was used as a positive control [38,42]. To test for expression of *APETALA3-1 (AqAP3-1), APETALA3-2 (AqAP3-2), APETALA3-3 (AqAP3-3),* and *FUL-like- 1 (AqFL1*) in VIGS-treated leaves, cDNA from several leaves were pooled together prior to amplification. The control pool consisted of *AqANS-*silenced control leaves C1-4, the *AqFIE* VIGS-treated pool consisted of *AqFIE* leaves F3-6, and the *AqEMF2* VIGS-treated pool consisted of *AqEMF2* leaves E1-4.

### qRT-PCR

cDNA was prepared from VIGS-treated tissue as described above. For the carpel sample, carpels were collected from 3 anthesis stage wild type plants and pooled together. RNA was extracted using the RNeasy Mini Kit (Qiagen, Valencia, CA) and treated as described above. cDNA from VIGS-treated tissue was then pooled together and diluted 1:10. The control sepal pool consisted of *AqANS-*silenced control sepals C1-4, the control petal pool consisted of *AqANS-*silenced control petals C1-4, the *AqFIE* sepal pool consisted of *AqFIE* VIGS-treated sepals F2, 3, 5, and 6, the *AqFIE* petal pool consisted of *AqFIE* VIGS-treated petals F2, 3, 5, and 6, the *AqEMF2* sepal pool consisted of *AqEMF2* VIGS-treated sepals E2, 3, and 4 s, and the *AqEMF2* petal pool consisted of *AqEMF2* VIGS-treated petals E1 and E2. qRT-PCR was performed using PerfeCTa qPCR FastMix, Low ROX (Quant Biosciences Inc., Gaithersburg, MD) in the Stratagene Mx3005P QPCR system to study the relative expression of *AqAG1* and *AqAG2. AqIPP2* expression was used for value normalization. All primers are listed in Additional file 1.

### Microscopy

Petals from wild type, *AqANS* VIGS-treated, and *AqEMF2* VIGS-treated plants were stored at -80°C and then warmed to room temperature and mounted whole on glass slides in water. Cells were visualized in the Harvard Center for Biological Imaging on a Zeiss AxioImager Z2 microscope using trans-illumination with white light. Images were taken using a Zeiss AxioCam Mrc digital camera.

## Results

We treated both unvernalized and vernalized plants with TRV2 constructs containing either *AqANS-AqFIE* or *AqANS-AqEMF2* fragments. TRV2-*AqANS* treated plants were used as controls throughout. Phenotypes of *AqFIE* and *AqEMF2* silenced plants were equivalent and will be discussed together. We also treated a small number of unvernalized plants with *AqANS-AqCLF* and *AqANS-AqSWN* VIGS constructs. Phenotypes from these plants were similar to those seen in *AqFIE* and *AqEMF2,* but were weaker (data not shown), most likely due to partial redundancy between *AqCLF* and *AqSWN.* Thus we chose to focus on *AqFIE* and *AqEMF2* VIGS-treated tissue. As is common for VIGS-treated plants, we recovered a range of phenotypes in a small percentage of VIGS-treated plants (roughly 10-15% of plants in each round) [36]. In the current experiment there is the added component that phenotypes are likely due to mis-expression of PRC2 target genes, and are therefore likely to have an added complexity due to ectopic expression of a potentially wide range of target loci.

### Vegetative phenotypes

**Figure 1:**
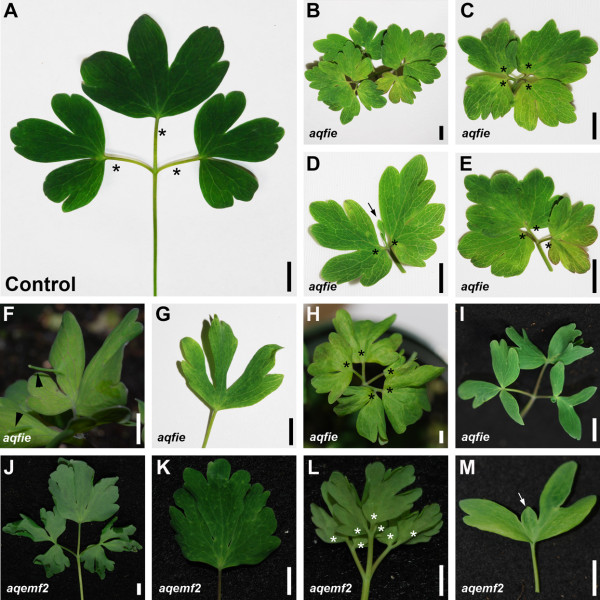
**Vegetative phenotypes of PRC2 VIGS-treated plants. A.***AqANS*-treated leaf (Control) with three lobed leaflets. First order petiolules are marked with asterisks. **B-I.***AqFIE*-silenced leaves and leaflets (abbreviated *aqfie*). **B.** Entire leaf with highly branched leaflets. **C-E.** Each leaflet from the leaf shown in B with higher order petiolules marked with asterisks and reduced central lobe indicated with an arrow. Leaflets are arranged in clockwise order starting with the left lateral leaflet in B. **F.** Leaflet with curled laminae, increased branching (asterisks) and ectopic outgrowth on the adaxial lamina (white arrowhead). **G.** Leaflet with reduced lamina and narrow lobes that are deeply divided. **H.** Entire leaf showing increasing internal branching (asterisks) and curling. **I.** Entire leaf with deep lobes and aberrantly shaped laminae. **J-M.***AqEMF2*-silenced leaves (abbreviated *aqemf2*). **J.** Entire leaf showing curled/ruffled laminae and deep lobing. **K.** Central leaflet from J exhibiting curled laminae, increased degree of lobing and serration. **L.** Entire leaf with internal branching (asterisks) and curled laminae. **M.** Leaflet with reduced central lobe (arrow). Scale bars: 1 cm.

Wild type *Aquilegia* leaves are compound, typically bearing three leaflets that are themselves divided into two to three lobes (Figure 1A). Although these leaflets are often relatively deeply lobed, they do not generally produce elongated, higher order petiolules within the leaflets. However, *A. x coerulea* does display heteroblasty over the course of its lifespan, varying leaf morphology as the individual progresses from the vegetative to the reproductive stage (Additional file 2). In late reproductive adult stages, higher order petiolules may be observed in which the central lobe of each leaflet becomes itself a separate leaflet borne its own petiolule (Additional file 2C). Using the terminology of Kim et al. 2003 [32], all of these leaf forms are non-peltately palmate in that the leaflets are not radially positioned around the terminus of the primary petiole.

We observed *AqANS* silencing in 10-15% of treated plants across the *AqFIE*- and *AqEMF2*-VIGS experiments. In addition to the *AqANS*-silencing, the leaves of these plants showed a complex set of phenotypes. The most consistently observed perturbation was curled or ruffled laminae that typically curled toward the abaxial surface (~10-12% of treated plants and, thus, the majority of silenced plants) (Figure 1F, H, J-L). We also observed an increased frequency of higher order branching in which fully formed petiolules developed within the leaflet, creating as many as ten or twelve distinct leaflets rather than the usual three (Figure 1B-F, H, L and Additional file 2E). While we have never observed such higher order branching in control leaves, either in the context of these experiments or others [40], we obtained 15 leaves from a total of 10 *AqFIE-* and *AqEMF2*-treated plants that exhibited increased branching. When quantified (Additional file 2E), the presence of higher order petiolules is significant at p < 0.05 for unvernalized lateral leaflets but not significant for the other stages/leaflet types. However, it is obvious that there is much more branching variation in silenced leaflets than in controls. In many cases, the margins of the laminae had additional lobing relative to control leaves (observed in 10% of treated plants) (Figure 1B-E, K) and, in a small number of cases, the central lobe of the terminal leaflet was severely reduced (seen in multiple leaves from 5 plants) (Figure 1D, M). Laminar area was highly variable with some leaflets appearing to have expanded area (~25 plants) (Figure 1F) while others seemed reduced (~8 plants) (Figure 1G, I, M). In two plants, ectopic finger-like projections were observed on the adaxial surface of laminae (Figure 1F), which was never observed in control leaflets.

### Floral phenotypes

**Figure 2:**
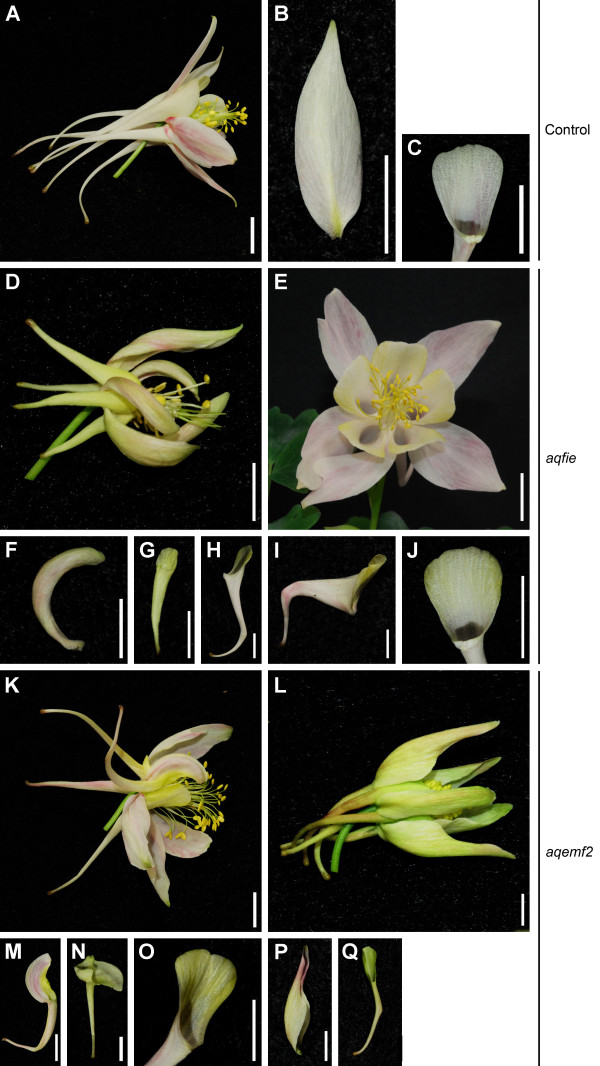
**Floral phenotypes of PRC2 VIGS-treated plants. A-C.***AqANS*-silenced control flower and perianth organs (Control). **A.** Entire flower. **B.** Entire sepal. **C.** Petal limb. **D-J.***AqFIE*-silenced flowers and organs (abbreviated *aqfie*). **D.** Severely affected flower. **E.** Moderately affected flower. **F.** Narrow, folded sepal of flower in D. **G.** Narrow, stunted petal of flower in D. **H-I.** Petals with bent spurs from moderately affected flowers. **J.** Yellow limb of moderately affected petal. **K-Q.***AqEMF2*-silenced flowers and organs (abbreviated *aqemf2*). **K-L.** Severely affected flowers. **M-N.** Sepal/petal chimeras from first whorl of flowers such as K. **O.** Yellow limb of second whorl petal from flower in K. **P.** Narrow, folded sepal from flower in L. **Q.** Narrow, bent petal from flower in L. Scale bars: 1 cm.

Wild type *A. x coerulea* flowers possess five organ types: sepals, petals, stamens, staminodia and carpels [39]. We have focused on the sepals and petals because they showed strong phenotypes in the silenced flowers. Wild type sepals are flat and ovate with an entire margin (Figure 2A-B). The petals are notable for the presence of a long hollow nectar spur, which forms near the attachment point (Figure 2A). This feature divides the organ into two regions, the proximal spur and the distal limb. Spurs in *A. x coerulea* are typically 5-6 cm in length and slightly curved. The limb region is relatively flat with a rounded, weakly lobed margin (Figure 2C). In 25 flowers from vernalized *AqFIE-* and *AqEMF2-*treated plants, we observed sepals that were narrower than wildtype organs and dramatically folded towards the adaxial surface (Figure 2D, F, L, P). In severely affected flowers, petals were narrowed and stunted (10 flowers, Figure 2D, G, Q) or exhibited sharply bent spurs (12 flowers, Figure 2H-I, K, M, Q). In two *AqEMF2*-silenced flowers, the sepals exhibited chimeric petal identity including ectopic spur formation (Figure 2M-N). Perhaps most surprising, many of the perianth organs had a definite yellow hue, with the petal limbs showing particularly intense yellow coloration (observed in at least one organ from 15 flowers) (Figure 2E, I-M, O). Such coloration was not observed in *AqANS*-silenced control flowers (Figure 2A-C). Examination of the *AqFIE*- and *AqEMF2*-silenced organs under high magnification reveals that yellow pigment is deposited in plastids (Additional file 3A), consistent with carotenoids rather than the vacuole-based aurones that are produced in some *Aquilegia* species [43,44].

### Assessment of AqFIE and AqEMF2 down-regulation

**Figure 3:**
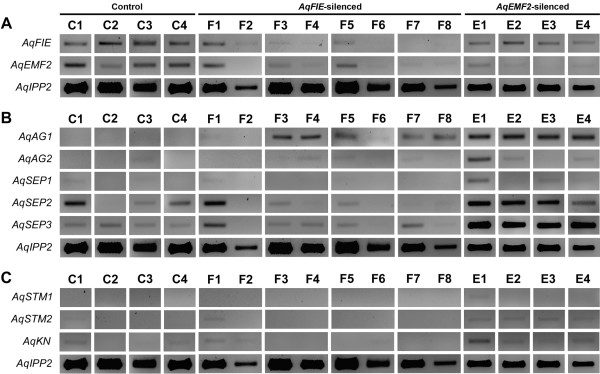
**RT-PCR expression data in PRC2 VIGS-treated leaves.***AqIPP2* was used as a loading control for all reactions. Note that the expression of *AqFIE* and *AqEMF2* are low relative to the expression of *AqIPP2*. **A.** Expression of *AqFIE* and *AqEMF2* in *AqANS-*silenced control leaves (C1-C4), and *AqFIE-*silenced (F1-F8) and *AqEMF3-*silenced (E1-E4) leaves. *AqFIE* is clearly down-regulated in *AqFIE-*silenced tissue and, likewise, *AqEMF2* is down-regulated in *AqEMF2-*silenced tissue. Interestingly, *AqEMF2* also appears to be down-regulated in *AqFIE*-treated leaves but *AqFIE* expression is unaffected in *AqEMF2-*treated leaves. **B.** Expression of several floral organ identity genes in *AqANS-*silenced control leaves (C1-C4), and *AqFIE-*silenced (F1-F8) and *AqEMF3-*silenced (E1-E4) leaves. In several of the *AqFIE* down-regulated leaves and all of the *AqEMF2* down-regulated leaves, *AqAG1* is over-expressed compared to *AqANS-*silenced control leaves. While the expression of the *SEPALLATA* homologs is variable in both control and experimental leaves, *AqSEP3* may be up-regulated in some of the *AqFIE-* and all of the *AqEMF2-silenced* leaves. **C.** Expression of several of the *A. x coerulea* class I KNOX genes in *AqANS-*silenced control leaves (C1-C4), and *AqFIE*-silenced (F1-F8) and *AqEMF3-*silenced (E1-E4) leaves. Expression of these genes is unaffected in the mature *AqFIE-* and *AqEMF2-*silenced leaves.

**Figure 4:**
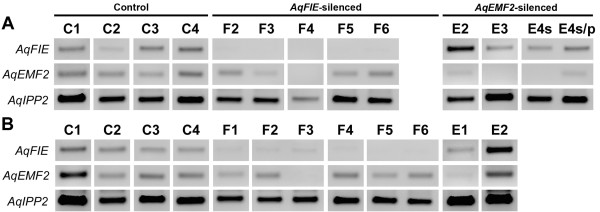
**Expression of *AqFIE *and *AqEMF2 *in VIGS-treated floral organs. A.** Expression of *AqFIE* and *AqEMF2* in *AqANS-*silenced control sepals (C1-C4), and *AqFIE-* (F2-F6) and *AqEMF3-* (E1-E4s/p) treated first whorl organs. *AqFIE* is down-regulated in all of the *AqFIE-*treated sepals. Likewise, *AqEMF2* is down-regulated in *AqEMF2-*treated first whorl organs. Unlike the pattern in leaves, *AqEMF2* is not down-regulated in *AqFIE*-treated sepals. **B.** Expression of *AqFIE* and *AqEMF2* in *AqANS-*silenced control petals (C1-C4), and *AqFIE-* (F1-F6) and *AqEMF2-* (E1 and E2) treated petals. *AqFIE* is down-regulated in all of the *AqFIE-*treated petals while *AqEMF2* is down-regulated in E1 and also in F3.

Due to limited RNA availability, we used standard RT-PCR to assess target gene down-regulation in leaves, sepals and petals compared to their expression in *AqANS* silenced control tissue. Even in *AqANS* silenced (control) tissue, *AqFIE* and *AqEMF2* are expressed at low levels relative to the loading control *AqIPP2.* The experimental samples (F1-8 and E1-4) were selected to represent all of the observed phenotypes and were derived from separate plants. This analysis demonstrated that in the TRV2-*AqFIE-AqANS* treated plants, *AqFIE* was strongly down-regulated, being undetectable in a number of samples (Figure 3A and Figure 4). Likewise, *AqEMF2* expression is reduced to undetectable levels in most tested *AqEMF2*-silenced samples (Figure 3A and Figure 4). We also tested for *AqEMF2* in *AqFIE*-treated plants and vice versa, and found that *AqEMF2* levels are often reduced in *AqFIE*-treated leaves, although the reciprocal is generally not true (Figure 3A). Furthermore, we tested the other PRC2-complex members, *AqCLF* and *AqSWN*, and found no consistent evidence of their down-regulation in either type of silenced tissue (Additional file 4A).

### Assessment of candidate gene expression

**Figure 5:**
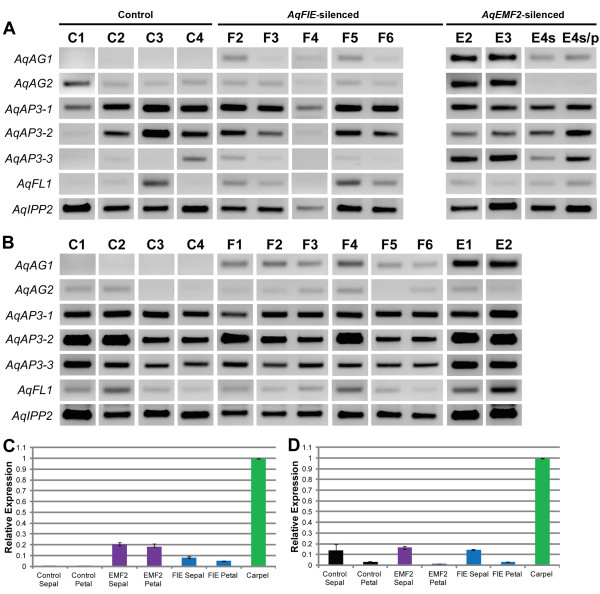
**Expression of candidate genes in PRC2 VIGS-treated floral organs. A.** Expression of several floral organ identity genes in *AqANS-*silenced control sepals (C1-C4), and *AqFIE-* (F2-F6) and *AqEMF2-* (E1-E4s/p) treated first whorl organs. *AqAG1* is up-regulated in all *AqFIE-* and *AqEMF2-*treated organs compared to the controls.*AqAP3-3* also appears to be up-regulated in some of the sepals, particularly in *AqEMF2* down-regulated first whorl organs, several of which were in fact sepal/petal chimeras (s/p). Expression of *AqAP3-2* and *AqFL1* is variable in mature sepals and is difficult to assess. *AqAG2* and *AqAP3-1* expression does not appear to be affected in silenced tissue. **B.** Expression of several floral organ identity genes in *AqANS-*silenced control petals (C1-C4), and *AqFIE-* (F2-F6) and *AqEMF2-* (E1-E4s/p) VIGS-treated petals. *AqAG1* is up-regulated in all *AqFIE-* and *AqEMF2-*treated tissue compared to the controls. **C** and **D.** Quantitative Real Time PCR analysis of expression of *AqAG1* and *AqAG2* in *AqFIE, AqEMF2,* and *AqANS* control silenced tissue and wild type carpels*.* cDNA from two to four samples was pooled together prior to analysis. For each data point, three technical replicates were analyzed. *AqIPP2* expression was used for normalization. **C.** Average fold change in the expression of *AqAG1* in *AqFIE, AqEMF2,* and *AqANS* control silenced tissue normalized to wild type carpels with SD error bars. **D.** Average fold change in the expression of *AqAG2* in *AqFIE, AqEMF2,* and *AqANS* control silenced tissue normalized to wild type carpels with SD error bars.

We tested for ectopic expression of a wide panel of potential target genes, with a focus on the floral organ identity loci and type I KNOX homologs (Figure 3, Figure 5, and Additional file 4B). We again compared the expression of these genes to expression in *AqANS-*silenced control tissue. One of the two *A. x coerulea* the C class MADS box genes, *AGAMOUS 1* (*AqAG1*) (see Additional file 1 for all gene identification numbers), is consistently up-regulated in silenced leaves and floral organs. The second *AGAMOUS* homologs, *AGAMOUS2* (*AqAG2*) may also be slightly up-regulated in some of the leaves, although *AqAG2* shows basal expression in control floral organs (Figures 3 and 5). The three *A. x coerulea SEPALLATA* paralogs (*AqSEP1, AqSEP2,* and *AqSEP3*) are somewhat difficult to assess because they are variably expressed in control leaves but *AqSEP3* in particular seems to be up-regulated in *AqEMF2*-silenced leaves (Figure 3B). These genes were not assessed in floral organs because they are already broadly expressed in these tissues. *A. x coerulea* also has three paralogs of the B class MADS box gene, *APETALA3* (*AqAP3-1, AqAP3-2,* and *AqAP3-3*). The petal-specific *AqAP3-3* locus is highly up-regulated in *AqEMF2*-silenced sepals, which also showed chimeric sepal/petal identity in several cases (Figure 5A). Additionally two of the three *AP3* paralogs are moderately up-regulated in PRC2 VIGS-treated leaves (Additional file 4B), but the expression of *AqAP3-1* and *AqAP3-2* is unaffected in mature sepals and petals (Figure 5A and B). We also looked at the expression of *FUL-like 1* (*AqFL1*), which is normally expressed in early leaves, but no ectopic expression was detected (Figure 5 and Additional file 4B).

Next, we tested for up-regulation of three of the five *A. x coerulea* class I KNOX genes. No significant ectopic KNOX gene expression could be detected in the leaves. Weak expression of the *SHOOTMERISTEMLESS* 2 (*AqSTM2*) and *KNOTTED* (*AqKN*) homologs is detected in *AqEMF2*-silenced leaves (Figure 3C), however, we also occasionally detected comparable expression of these genes in control (*AqANS-*silenced) leaves, so it is difficult to ascribe significance to this expression. Given the lack of clear up-regulation in leaves and due to a limited amount of floral RNA, class I KNOX gene expression was not tested in the floral organs.

Although *AqAG1* is consistently over-expressed in *AqFIE* and *AqEMF2* silenced sepals and petals, we never saw any evidence of carpel identity in these organs. We therefore pooled cDNA from several *AqANS-* (control)*, AqFIE-,* and *AqEMF2-*treated petals and sepals and used qRT-PCR to further examined the expression of *AqAG1* and *AqAG2* in these organs as well as in wild type carpels (Figure 5C and D). We found that while *AqAG1* was clearly up-regulated in *AqFIE* and *AqEMF2* silenced organs compared to the controls, *AqAG1* expression was still much lower than in wild type carpels (about 0.05 to 0.2 fold). In contrast, *AqAG2* expression was similar in control and PRC2 silenced tissue, but much lower than in wild type carpels.

Lastly, in an effort to investigate the carotenoid production, we identified the likely *A. x coerulea* homologs of a range of components of the carotenoid pathway in *A. thaliana*, including enzymes involved in production (*PHYTOENE SYNTHASE* (*PSY*) and *CAROTENOID ISOMERASE* (*CRTISO*)) and breakdown (*CAROTENOID CLEAVAGE DIOXYGENASE 4* (*CCD4*) and *9-CIS-EPOXYCAROTENOID DIOXYGENASE 3* (*NCED3*)) of carotenoids [45]. *A. x coerulea* has two copies *CCD4* (*AqCCD4* and *AqCCD4L)* and two genes that are closely related to *A. thaliana PSY* (*AqPSYL1* and *AqPSYL2*). Previous studies in *A. thaliana* have indicated that both *CRTISO* and *NCED3* are positively epigenetically regulated by other SET domain containing proteins so we were particularly interested in the expression of these genes in *AqFIE* and *AqEMF2* down-regulated tissue [46,47]. We used RT-PCR to examine the expression of these six genes in *AqANS-* (control), *AqFIE-,* and *AqEMF2-*treated petals (Additional file 3B). Given the observed phenotypes, we might expect the expression of *AqPSYL1*, *AqPSYL2*, or *AqCRTISO* to be up-regulated or *AqCCD4, AqCCD4L*, or *AqNCED3* to be down-regulated. Unfortunately, no clear patterns are apparent from these reactions.

## Discussion

*AqFIE* and *AqEMF2* VIGS-treated plants displayed a range of lateral organ phenotypes. Silenced leaves often had ruffled or curled lamina, additional lobing, and an increased frequency of higher order branching. The perianth organs were generally narrower than wild type organs. Sepals were also curled and petals were stunted or had bent spurs, while petal limbs also had a particularly intense yellow coloration seemingly due to an accumulation of carotenoid pigments in these cells. Many of the phenotypes we observed are similar to those seen in *clf* mutants and *FIE* cosuppressed *A. thaliana*, including curled leaves and narrow perianth organs [17,29]. Unlike *clf* mutants and *AG* over-expressers in *A. thaliana,* dramatic transformation towards carpel identity was not observed in the *AqFIE-* and *AqEMF2-*treated sepals or petals. However, the level of *AqAG1* expression in these organs was much less than what is seen in wild type *Aquilegia* carpels. Interestingly, the distinct folded morphology of the sepals may suggest slight transformation towards carpel identity as silenced leaves were folded towards the abaxial surface while the sepals were dramatically folded towards the adaxial surface, which is similar to the folding pattern of the *Aquilegia* carpel [48].

It is interesting to note that in *AqFIE* silenced leaves, *AqEMF2* is also down-regulated. The reverse is not true in *AqEMF2* silenced leaves, and *AqEMF2* expression is not affected in *AqFIE* silenced floral organs. This result suggests that PRC2 may be directly or indirectly regulating *AqEMF2* expression in *A. x coerulea* leaves, which could account for the generally more severe phenotypes observed in *AqFIE* silenced leaves compared to *AqEMF2* silenced leaves. *AqEMF2* is the only member of the complex that appears to be PRC2-regulated as the expression of *AqCLF* and *AqSWN* is not affected in PRC2 down-regulated leaves. In general, the potential for this type of cross-regulation is relatively unexplored in *A. thaliana* and, therefore, bears further study.

In our analysis of candidate target genes, we found that *AqAG1* is often ectopically expressed in PRC2 down-regulated tissue. *AqAP3-3* and *AqSEP3* are also up-regulated in some organs, but expression of the class I KNOX genes and several candidate genes involved in carotenoid production or degradation seem largely unaffected. Mutations in *AG* and *SEP3* are known to suppress the curled leaf phenotype in *clf* mutant plants while over-expression of these MADS box genes, which themselves function together in a complex [49], is thought to be the cause of the curled leaf phenotype [26]. It is, therefore, possible that over-expression of *AqAG1* and *AqSEP3* is similarly responsible for many of the observed phenotypes in *AqFIE* and *AqEMF2* silenced leaves. These findings lead us to conclude that PRC2-based regulation of *AG* and *SEP3* homologs is deeply conserved in eudicots. It has recently been shown that several chromatin remodeling factors associate with MADS complexes and one model is that an important function of MADS domain complexes may be to recruit chromatin remodeling complexes to target loci in order to alter transcription of these genes and direct organ development [50,51]. For example, RELATIVE OF EARLY FLOWERING 6 (REF6) was enriched in protein complexes that were isolated via immunoprecipitation using tagged ABCE class MADS box proteins [50]. REF6 has been shown to specifically demethylate H3K27me3, the histone modification deposited by PRC2 [52]. Activation of *SEP3* by *APETALA1* (*AP1*) in *A. thaliana* results in the reduction of H3K27me3 at the *SEP3* promoter, suggesting that *AP1* may recruit *REF6* to the *SEP3* promoter in order to help induce *SEP3* gene function [50]. Our data suggests that this key dependency on epigenetic regulation for the switch from vegetative to floral development may be important outside of *A. thaliana*. There are some complications, however. Of the two *A. x coerulea AG* homologs, only one, *AqAG1*, is strongly regulated by PRC2. Perhaps consistent with this observation, sequencing of the *Aquilegia* genome (http://www.phytozome.net/search.php?method=Org_Acoerulea) reveals that *AqAG1* does contain the large regulatory second intron that is common to *AG* homologs [53,54] while *AqAG2*’s second intron is much smaller. These results suggest that PRC2 regulation can be directed in a paralog-specific fashion and may even play some role in the distinct expression patterns observed among these gene copies [39].

The class I KNOX genes are directly or indirectly regulated by PRC2 in both *A. thaliana* and *Physcomitrella*, however, we detected little or no increase in KNOX gene expression in our *AqFIE* and *AqEMF2* silenced leaves. This is somewhat surprising because of the higher order branching that we observed in silenced leaves, including several of the tested RNA samples. The class I KNOX genes are thought to play a role in compound leaf development in a number of species. In many, but not all, compound leafed taxa where KNOX gene expression has been studied, including *Aquilegia*, it has been shown that the genes are expressed in the shoot apical meristem and down-regulated in incipient leaf primordia (P0), but subsequently turned back on in early leaf primordia [28]. Down-regulation of class I KNOX genes in the leaves of models such as tomato or *Cardamine* causes reduced branching while over-expression leads to increased branching [55,56], suggesting that KNOX genes act to maintain indeterminacy in compound leaves and promote leaflet initiation.

There are several possible explanations for why we did not observe significant ectopic KNOX gene expression in our VIGS-treated leaves. First, it is possible the KNOX genes were ectopically expressed early in leaf development when the higher order branching actually developed, but were later down-regulated by redundant mechanisms, such as *ASYMMETRIC LEAVES 1* (*AS1*)-mediated repression [57,58]. In *A. thaliana AS1* mediated silencing of some of the KNOX genes has been shown to require the PRC2 complex and it is thought that AS1 and AS2 directly recruit the PRC2 complex to KNOX loci [59]. However, it is important to remember that in other taxa with compound leaves, the KNOX and *AS1* homologs have lost their mutually exclusive regulatory interactions and are expressed together at later stages [32]. This may suggest that the *AS1*-dependent epigenetic silencing of KNOX genes that has been described in several simple-leafed models [57,58] does not hold for plants with compound leaves. Along these lines, it is also possible that the increased branching phenotypes are due to other factors, such as accelerated phase change or novel genetic mechanisms regulating leaflet branching in *Aquilegia*. For instance, a recent functional study of the gene *AqFL1* in *A. x coerulea* revealed that it promotes proper leaf margin development, a unique finding for homologs of this gene lineage [40]. This raises the possibility that factors other than the KNOX genes contribute to compound leaf branching in *Aquilegia*.

In addition to the conserved role in regulating *AG, AP3,* and *SEP3, A. x coerulea* PRC2 may target novel pathways, including those regulating carotenoid production or degradation. In *A. thaliana* patches of yellow anther-like tissue are observed on *clf* mutant petals [17]. However, the yellow pigmentation we observed is due to the accumulation of carotenoids in the plastids rather than to a partial homeotic transformation. While genes in the carotenoid pathway are not known to be suppressed by PRC2, some loci are positively epigenetically regulated in *A. thaliana*. Previous studies have shown that a major enzyme in the carotenoid biosynthesis pathway, CRTISO, requires the chromatin modifying enzyme SET DOMAIN GROUP 8 (SDG8) to maintain its expression [46]. NCED3, an enzyme that cleaves some types of carotenoids as a part of abscisic acid (ABA) synthesis, is similarly epigenetically regulated by the *A. thaliana* trithorax homolog ATX1 [47]. While none of the genes we tested were consistently up- or down-regulated in *AqFIE* and *AqEMF2* silenced petals, carotenoid production is very genetically complex and we were unable to test all of the candidate loci [60]. Thus, it seems likely that PRC2 regulates an as yet unidentified enzyme in this pathway in *A. x coerulea.*

## Conclusions

• A critical role for PRC2 in maintaining the repression of *AG*, *SEP3*, and possibly *AP3* appears to be conserved across eudicots. This conservation underscores the importance of chromatin remodeling factors in regulating the floral transition and the proper localization of floral organ identity.

• Class I KNOX genes are not ectopically expressed in PRC2 down-regulated tissue in *A. x coerulea*, possibly due to a regulatory shift associated with the evolution of compound leaves.

• *A. x coerulea* PRC2 plays a significant role in regulating the carotenoid pathway in floral organs, which has not been observed in other taxa.

• This study, the first to examine PRC2 function in angiosperms outside *A. thaliana* or the grasses, highlights how little we still know about the general conservation or targeting mechanisms underlying PRC2 function in major developmental transitions.

## Abbreviations

AG: *AGAMOUS*; ANS: *Anthocyanin synthase*; AP1: *APETALA1*; AP3: *APETALA3*; Aq: *Aquilegia*; BP: *Brevipedicellus*; bp: Base pair(s); CCD: *CAROTENOID CLEAVAGE DIOXYGENASE*; cDNA: DNA complementary to RNA; CLF: *CURLY LEAF*; cm: Centimeter; CRTISO: *CAROTENOID ISOMERASE*; DNA: Deoxyribonucleic acid; DNase: Deoxyribonuclease; E(z): *Enhancer of zeste*; EMF2: *EMBRYONIC FLOWER 2*; ESC: *Extra sex combs*; Eudicots: Eudicotyledonous; FIE: *FERTILIZATION INDEPENDENT ENDOSPERM*; FIS: *FERTILIZATION INDEPENDENT SEED*; FLC: *FLOWERING LOCUS c*; FL1: *FRUITFUL-like 1*; H3K27: Histone H3 Lysine 27; HOX: Homeobox; IPP2: *Isopentyl pyrophosphate:dimethylallyl pyrophosphate isomerase*; KN: *KNOTTED*; KNAT2: *KNOTTED-like from Arabidopsis thaliana 2*; KNOX: *knotted1* homeobox gene; MADS: *MCM1, agamous, deficiens, SRF*; MEA: *Medea*; MKN: *Moss knotted1-like*; MSI1: *Multi copy suppressor of IRA 1*; n: Chromosome number; NCED: *9-CIS-epoxycarotenoid dioxygenase*; oligo: Oligodeoxyribonucleotide; PcG: Polycomb group; Pp: *Physcomitrella patens*; PCR: Polymerase chain reaction; PRC1: Polycomb repressive complex1; PRC2: Polycomb repressive complex2; PSY: *Phytoene Synthase*; qRT-PCR: Quantitative real time polymerase chain reaction; REF6: Relative of early flowering 6; RNA: Ribonucleic acid; RT-PCR: Reverse transcriptase PCR; SEP: *SEPALLATA*; STM: *SHOOTMERISTEMLESS*; Su(z)12: *Suppressor of zeste12*; SWN: *SWINGER*; TRV: Tobacco rattle virus; VIGS: Virus-induced gene silencing.

## Competing interests

The authors declare that they have no competing interests.

## Authors’ contributions

EJG helped to conceive of the study, carried out the experiments, and drafted the manuscript. EMK helped to conceive of the study, supervised the experiments, and helped draft the manuscript. Both authors read and approved the final manuscript.

## Supplementary Material

Additional file 1 Table of all PCR primers. Click here for file

Additional file 2 **Heteroblasty in *A. x coerulea* leaves.** A. Unvernalized leaf with 3 major lobes in the lateral leaflets. B. Unvernalized leaf with 2 major lobes in the lateral leaflets. C. Vernalized leaf with higher order petiolules where the central lobe of each leaflet is a separate leaflet borne on a petiolule (asterisks). D. Vernalized leaf with 2 major lobes in the lateral leaflets. These leaves are more deeply lobed than similar unveralization leaves. E. Average number of higher order petiolules within medial or lateral leaflets in wild type unvernalized, *AqFIE* silenced unvernalized, wild type vernalized, and *AqFIE* silenced leaves with standard deviations. Both unvernalized and vernalized *AqFIE* silenced lateral leaflets had on average more higher order petiolules than the wild type. Unvernalized *AqFIE* silenced lateral leaflets also had a slightly higher average number of higher order petiolules compared to wild type, but vernalized *AqFIE* silenced leaves had a slightly lower number of petiolules per medial leaflet. When quantified, this increase is significant (*) at p < 0.05 for unvernalized lateral leaflets but not significant for the other stages/leaflet types.3 Click here for file

Additional file 3 **The PRC2 regulates carotenoid production in *A. coerulea *** **petals.** A. High magnification views of epidermal cells in *A. x coerulea* petal limbs. From left to right: Anthocyanin of untreated petal limb (anthocyanin is deposited in the vacuole, resulting in a very even distribution of color), almost complete lack of color in *AqANS*-silenced petal limb, and punctate pattern of carotenoid deposition in plastids of *AqEMF2*-silenced petal limb. B. Expression of several *A. x coerulea* homologs of genes important in carotenoid production (*CRTISO* and *PSY*) and degradation (*CCD4* and *NCED3*) in *AqANS-*silenced control petals (C1-C4) and *AqFIE* (F1-F6) and *AqEMF2* (E1 and E2) treated petals. Petals with strong yellow pigment are highlighted in dark yellow (F1, F5, and E1) and petals with pale yellow pigment are highlighted in light yellow (F2-F4). The expression of these genes is not consistently affected in the *AqFIE* and *AqEMF2* silenced petal samples. It is possible that other genes in the carotenoid pathway are being misexpressed. Scale bars: 10 μm. Click here for file

Additional file 4 **Additional candidate gene expression in PRC2 VIGS-treated leaves.** A. Expression of *AqCLF* and *AqSWN* in *AqFIE-* and *AqEMF2-*treated leaves. Although *AqEMF2* appears to be down-regulated in some *AqFIE-*silenced leaves, the expression of *AqCLF* and *AqSWN* in these leaves is not affected. B. Expression of *AqAG1*, *AqFL1, AqAP3-1, AqAP3-2,* and *AqAP3-3* in pooled *AqANS* silenced control leaves (C) and *AqFIE* (F) and *AqEMF2* (E) silenced leaves. *AqAP3-1 AqAP3-2* and *AqAP3-3* is moderately up-regulated in both *AqFIE* and *AqEMF2* silenced tissue while *AgFL1* expression is unaffected. Click here for file

## References

[B1] Holliday R Epigenetics: an overview Dev Gen 1994 15 6 453 457 10.1002/dvg.1020150602 7834903

[B2] Russo VEA Martienssen R Riggsm AD Epigenetic Mechanisms of Gene Regulation 1996 Woodbury, NY, USA: Cold Spring Harbor Laboratory Press

[B3] Feil R Epigenetics, an emerging discipline with broad implications C R Biol 2008 331 11 837 843 10.1016/j.crvi.2008.07.027 18940698

[B4] Lewis EB A gene complex controlling segmentation in Drosophila Nature 1978 276 565 570 10.1038/276565a0 103000

[B5] Hennig L Derkacheva M Diversity of Polycomb group complexes in plants: same rules, different players? Trends Genet 2009 25 9 414 423 10.1016/j.tig.2009.07.002 19716619

[B6] Sawarkar R Paro R Interpretation of developmental signaling at chromatin: The polycomb perspective Dev Cell 2010 19 5 651 661 10.1016/j.devcel.2010.10.012 21074716

[B7] Kohler C Hennig L Regulation of cell identity by plant Polycomb and trithorax group proteins Curr Opin Genet Dev 2010 20 5 541 547 10.1016/j.gde.2010.04.015 20684877

[B8] Schubert D Primavesi L Bishopp A Roberts G Doonan J Jenuwein T Goodrich J Silencing by plant Polycomb-group genes requires dispersed trimethylation of histone H3 at lysine 27 Embo Journal 2006 25 19 4638 4649 10.1038/sj.emboj.7601311 16957776 PMC1590001

[B9] Nekrasov M Wild B Muller J Nucleosome binding and histone methyltransferase activity of Drosophila PRC2 EMBO Rep 2005 6 4 348 353 10.1038/sj.embor.7400376 15776017 PMC1299286

[B10] Pien S Grossniklaus U Polycomb group and trithorax group proteins in Arabidopsis Biochimica Et Biophysica Acta-Gene Structure and Expression 2007 1769 5–6 375 382 10.1016/j.bbaexp.2007.01.010 17363079

[B11] Spillane C Schmid KJ Laoueille-Duprat S Pien S Escobar-Restrepo JM Baroux C Gagliardini V Page DR Wolfe KH Grossniklaus U Positive darwinian selection at the imprinted MEDEA locus in plants Nature 2007 448 7151 349 U348 10.1038/nature05984 17637669

[B12] Luo M Platten D Chaudhury A Peacock WJ Dennis ES Expression, imprinting, and evolution of rice homologs of the Polycomb group genes Mol Plant 2009 2 4 711 723 10.1093/mp/ssp036 19825651

[B13] Whitcomb SJ Basu A Allis CD Bernstein E Polycomb Group proteins: an evolutionary perspective Trends Genet 2007 23 494 502 10.1016/j.tig.2007.08.006 17825942

[B14] Kohler C Hennig L Spillane C Pien S Gruissem W Grossniklaus U The Polycomb-group protein MEDEA regulates seed development by controlling expression of the MADS-box gene PHERES1 Genes Dev 2003 17 12 1540 1553 10.1101/gad.257403 12815071 PMC196083

[B15] Yoshida N Yanai Y Chen L Kato Y Hiratsuka J Miwa T Sung ZR Takahashi S EMBRYONIC FLOWER2, a novel polycomb group protein homolog, mediates shoot development and flowering in Arabidopsis Plant Cell 2001 13 11 2471 2481 11701882 10.1105/tpc.010227 PMC139465

[B16] Gendall AR Levy YY Wilson A Dean C The VERNALIZATION2 gene mediates the epigenetic regulation of vernalization in Arabidopsis Cell 2001 107 525 535 10.1016/S0092-8674(01)00573-6 11719192

[B17] Goodrich J Puangsomlee P Martin M Long D Meyerowitz EM Coupland G A polycomb-group gene regulates homeotic gene expression in Arabidopsis Nature 1997 386 6620 44 51 10.1038/386044a0 9052779

[B18] Oliver SN Finnegan EJ Dennis ES Peacock WJ Trevaskis B Vernalization-induced flowering in cereals is associated with changes in histone methylation at the VERNALIZATION1 gene Proc Natl Acad Sci USA 2009 106 20 8386 8391 10.1073/pnas.0903566106 19416817 PMC2677093

[B19] Rodrigues J Luo M Berger F Koltunow A Polycomb group gene function in sexual and asexual seed development in angiosperms Sex Plant Reprod 2010 23 2 123 133 10.1007/s00497-009-0131-2 20039181

[B20] Okano Y Aono N Hiwatashi Y Murata T Nishiyama T Ishikawa T Kubo M Hasebe M A Polycomb Repressive Complex 2 gene regulates apogamy and gives evolutionary insights into early land plant evolution Proc Natl Acad Sci USA 2009 106 38 16321 16326 10.1073/pnas.0906997106 19805300 PMC2752547

[B21] Mosquna A Katz A Decker EL Rensing SA Reski R Ohad N Regulation of stem cell maintenance by the Polycomb protein FIE has been conserved during land plant evolution Development 2009 136 14 2433 2444 10.1242/dev.035048 19542356

[B22] Bowman J Smyth DR Meyerowitz E The ABC model of flower development: then and now Development 2012 139 22 4095 4098 10.1242/dev.083972 23093420

[B23] Bowman JL Drews GN Meyerowitz EM Expression of the Arabidopsis floral homeotic gene agamous is restricted to specific cell types late in flower development Plant Cell 1991 3 749 758 1726485 10.1105/tpc.3.8.749 PMC160042

[B24] Bowman JL Smyth DR Meyerowitz EM Genes directing flower development in Arabidopsis Plant Cell 1989 1 37 52 2535466 10.1105/tpc.1.1.37 PMC159735

[B25] Foronda D de Navas LF Garaulet DL Sanchez-Herrero E Function and specificity of Hox genes Int J Dev Biol 2009 53 8–10 1409 1419 10.1387/ijdb.072462df 19247930

[B26] Lopez-Vernaza M Yang SX Muller R Thorpe F de Leau E Goodrich J SEPALLATA3, FT and FLC genes as targets of the Polycomb group gene CURLY LEAF PLoS ONE 2012 7 2 e30715 10.1371/journal.pone.0030715 22363474 PMC3281876

[B27] Wagner D Chromatin regulation of plant development Curr Opin Plant Biol 2003 6 1 20 28 10.1016/S1369526602000079 12495747

[B28] Bharathan G Goliber TE Moore C Kessler S Pham T Sinha NR Homologies in leaf form inferred from KNOXI gene expression during development Science 2002 296 5574 1858 1860 10.1126/science.1070343 12052958

[B29] Katz A Oliva M Mosquna A Hakim O Ohad N FIE and CURLY LEAF polycomb proteins interact in the regulation of homeobox gene expression during sporophyte development Plant Journal 2004 37 5 707 719 10.1111/j.1365-313X.2003.01996.x 14871310

[B30] Singer SD Ashton NW Revelation of ancestral roles of KNOX genes by a functional analysis of Physcomitrella homologues Plant Cell Reports 2007 26 2039 2054 10.1007/s00299-007-0409-5 17724598

[B31] Maizel A Busch MA Tanahashi T Perkovic J Kato M Hasebe M Weigel D The floral regulator LEAFY evolves by substitutions in the DNA binding domain Science 2005 308 5719 260 263 10.1126/science.1108229 15821093

[B32] Kim M McCormick S Timmermans M Sinha N The expression domain of PHANTASTICA determines leaflet placement in compound leaves Nature 2003 424 438 443 10.1038/nature01820 12879073

[B33] Hodges SA Kramer EM Columbines Curr Biol 2007 17 23 R992 R994 10.1016/j.cub.2007.09.034 18054769

[B34] Kramer EM Hodges SA Aquilegia as a model system for the evolution and ecology of petals Philos Trans R Soc Lond B Biol Sci 2010 365 477 490 10.1098/rstb.2009.0230 20047874 PMC2838260

[B35] Kramer EM Aquilegia: A new model for plant development, ecology, and evolution Ann Rev Plant Biol 2009 60 261 277 10.1146/annurev.arplant.043008.092051 19575583

[B36] Gould B Kramer EM Virus-induced gene silencing as a tool for functional analyses in the emerging model plant Aquilegia (columbine, Ranunculaceae) Plant Methods 2007 3 6 10.1186/1746-4811-3-6 17430595 PMC1855323

[B37] Sharma B Kramer EM Sub- and neofunctionalization of APETALA3 paralogs have contributed to the evolution of novel floral organ identity in Aquilegia (columbine, Ranunculaceae) New Phytol 2013 197 949 957 10.1111/nph.12078 23278258

[B38] Sharma B Guo C Kong H Kramer EM Petal-specific subfunctionalization of an APETALA3 paralog in the Ranunculales and its implications for petal evolution New Phytol 2011 190 870 883 21557746 10.1111/j.1469-8137.2011.03744.x

[B39] Kramer EM Holappa L Gould B Jaramillo MA Setnikov D Santiago P Elaboration of B gene function to include the identity of novel floral organs in the lower eudicot Aquilegia (Ranunculaceae) Plant Cell 2007 19 3 750 766 10.1105/tpc.107.050385 17400892 PMC1867376

[B40] Pabón-Mora N Sharma B Holappa L Kramer EM Litt A The Aquilegia FRUITFULL-like genes play key roles in leaf morphogenesis and inflorescence development Plant J 2013 74 2 197 212 10.1111/tpj.12113 23294330

[B41] Gleason E Kramer EM Characterization of *Aquilegia* Polycomb Repressive Complex 2 homologs reveals absence of imprinting Gene 2012 10.1016/j.gene.2012.07.004 22796128

[B42] Ballerini ES Kramer EM The control of flowering time in the lower eudicot Aquilegia formosa EvoDevo 2011 2 4 10.1186/2041-9139-2-4 21329499 PMC3049749

[B43] Vishnevetsky M Ovadis M Vainstein A Carotenoid sequestration in plants: the role of carotenoid-associated proteins Trends Plant Sci 1999 4 6 232 235 10.1016/S1360-1385(99)01414-4 10366880

[B44] Ono E Hatayama M Isono Y Sato T Watanabe R Yonekura-Sakakibara K Fukuchi-Mizutani M Tanaka Y Kusumi T Nishino T Localization of a flavonoid biosynthetic polyphenol oxidase in vacuoles Plant J 2006 45 2 133 143 10.1111/j.1365-313X.2005.02625.x 16367960

[B45] Cazzonelli CI Carotenoids in nature: insights from plants and beyond Funct Plant Biol 2011 3 1 833 847 10.1071/FP11192 32480941

[B46] Cazzonelli CI Roberts AC Carmody ME Pogson BJ Transcriptional control of SET domain group 8 and carotenoid isomerase during Arabidopsis development Mol Plant 2010 3 1 174 191 10.1093/mp/ssp092 19952001

[B47] Ding Y Avramova Z Fromm M The Arabidopsis trithorax-like factor ATX1 functions in dehydration stress responses via ABA-dependent and ABA-independent pathways Plant J 2011 66 5 735 744 10.1111/j.1365-313X.2011.04534.x 21309869

[B48] Tucker SC Hodges SA Floral ontogeny of Aquilegia, Semiaquilegia, and Isopyrum (Ranunculaceae) Int J Plant Sci 2005 166 4 557 574 10.1086/429848

[B49] Honma T Goto K Complexes of MADS-box proteins are sufficient to convert leaves into floral organs Nature 2001 409 6819 525 529 10.1038/35054083 11206550

[B50] Smaczniak C Immink RGH Angenent GC Kaufmann K Developmental and evolutionary diversity of plant MADS-domain factors: insights from recent studies Development 2012 139 17 3081 3098 10.1242/dev.074674 22872082

[B51] Immink RGH Kaufmann K Angenent GC The ‘ABC’ of MADS domain protein behaviour and interactions Semin Cell Dev Biol 2010 21 1 87 93 10.1016/j.semcdb.2009.10.004 19883778

[B52] Lu FL Cui X Zhang SB Jenuwein T Cao XF Arabidopsis REF6 is a histone H3 lysine 27 demethylase Nat Genet 2011 43 7 715 719 10.1038/ng.854 21642989

[B53] Deyholos MK Sieburth LE Separable whorl-specific expression and negative regulation by enhancer elements within the AGAMOUS second intron Plant Cell 2000 12 10 1799 1810 11041877 10.1105/tpc.12.10.1799 PMC149120

[B54] Hong RL Hamaguchi L Busch M Weigel D Regulatory elements of the floral homeotic gene AGAMOUS identified by phylogenetic footprinting and shadowing Plant Cell 2003 15 1296 1309 10.1105/tpc.009548 12782724 PMC156367

[B55] Hareven D Gutfinger T Parnis A Eshed Y Lifschitz E The making of a compound leaf: genetic manipulation of leaf architecture in tomato Cell 1996 84 5 735 744 10.1016/S0092-8674(00)81051-X 8625411

[B56] Hay A Barkoulas M Tsiantis M ASYMMETRIC LEAVES1 and auxin activities converge to repress BREVIPEDICELLUS expression and promote leaf development in Arabidopsis Development 2006 133 20 3955 3961 10.1242/dev.02545 16971475

[B57] Guo M Thomas J Collins G Timmermans MCP Direct repression of KNOX loci by the ASYMMETRIC LEAVES1 complex of Arabidopsis Plant Cell 2008 20 1 48 58 10.1105/tpc.107.056127 18203921 PMC2254922

[B58] Phelps-Durr TL Thomas J Vahab P Timmermans MCP Maize rough sheath2 and its Arabidopsis orthologue ASYMMETRIC LEAVES1 interact with HIRA, a predicted histone chaperone, to maintain knox gene silencing and determinacy during organogenesis Plant Cell 2005 17 11 2886 2898 10.1105/tpc.105.035477 16243907 PMC1276017

[B59] Lodha M Marco CF Timmermans M The ASYMMETRIC LEAVES complex maintains repression of KNOX homeobox genes via direct recruitment of Polycob-repressive complex2 Genes Dev 2013 27 596 601 10.1101/gad.211425.112 23468429 PMC3613607

[B60] Lu S Li L Carotenoid metabolism: biosynthesis, regulation and beyond J Integr Plant Biol 2008 50 7 778 785 10.1111/j.1744-7909.2008.00708.x 18713388

