# Supplementary figures and images for: Conserved roles for Polycomb Repressive Complex 2 in the regulation of lateral organ development in Aquilegia x coerulea ‘Origami’
Source: BMC Plant Biol. 2013 Nov 20;13:185. doi: 10.1186/1471-2229-13-185 (PMC3840678; doi:10.1186/1471-2229-13-185)

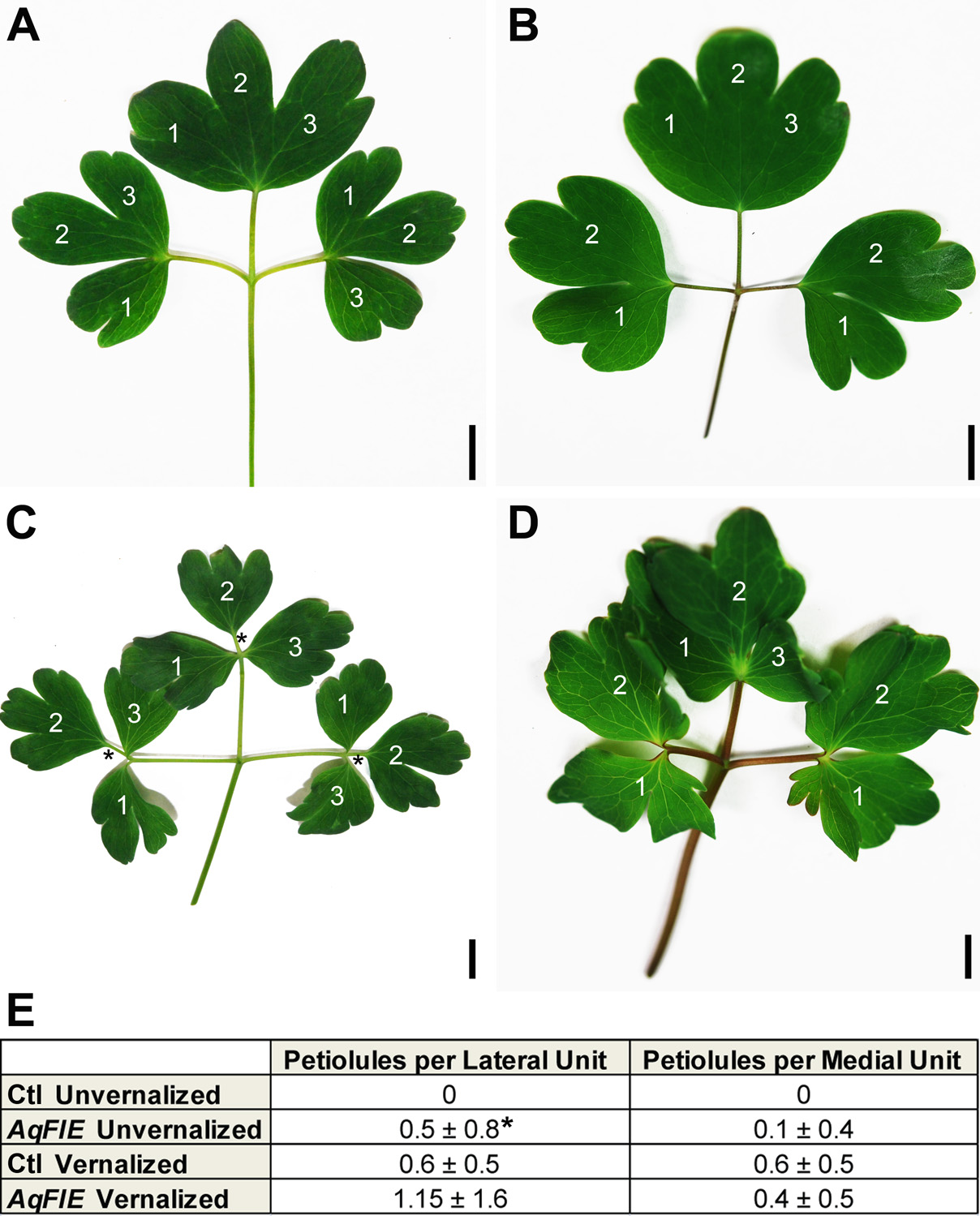

Supplement: Additional file 2 — Heteroblasty in A. x coerulea leaves. A. Unvernalized leaf with 3 major lobes in the lateral leaflets. B. Unvernalized leaf with 2 major lobes in the lateral leaflets. C. Vernalized leaf with higher order petiolules where the central lobe of each leaflet is a separate leaflet borne on a petiolule (asterisks). D. Vernalized leaf with 2 major lobes in the lateral leaflets. These leaves are more deeply lobed than similar unveralization leaves. E. Average number of higher order petiolules within medial or lateral leaflets in wild type unvernalized, AqFIE silenced unvernalized, wild type vernalized, and AqFIE silenced leaves with standard deviations. Both unvernalized and vernalized AqFIE silenced lateral leaflets had on average more higher order petiolules than the wild type. Unvernalized AqFIE silenced lateral leaflets also had a slightly higher average number of higher order petiolules compared to wild type, but vernalized AqFIE silenced leaves had a slightly lower number of petiolules per medial leaflet. When quantified, this increase is significant (*) at p < 0.05 for unvernalized lateral leaflets but not significant for the other stages/leaflet types.3 [file 1471-2229-13-185-S2.jpeg]

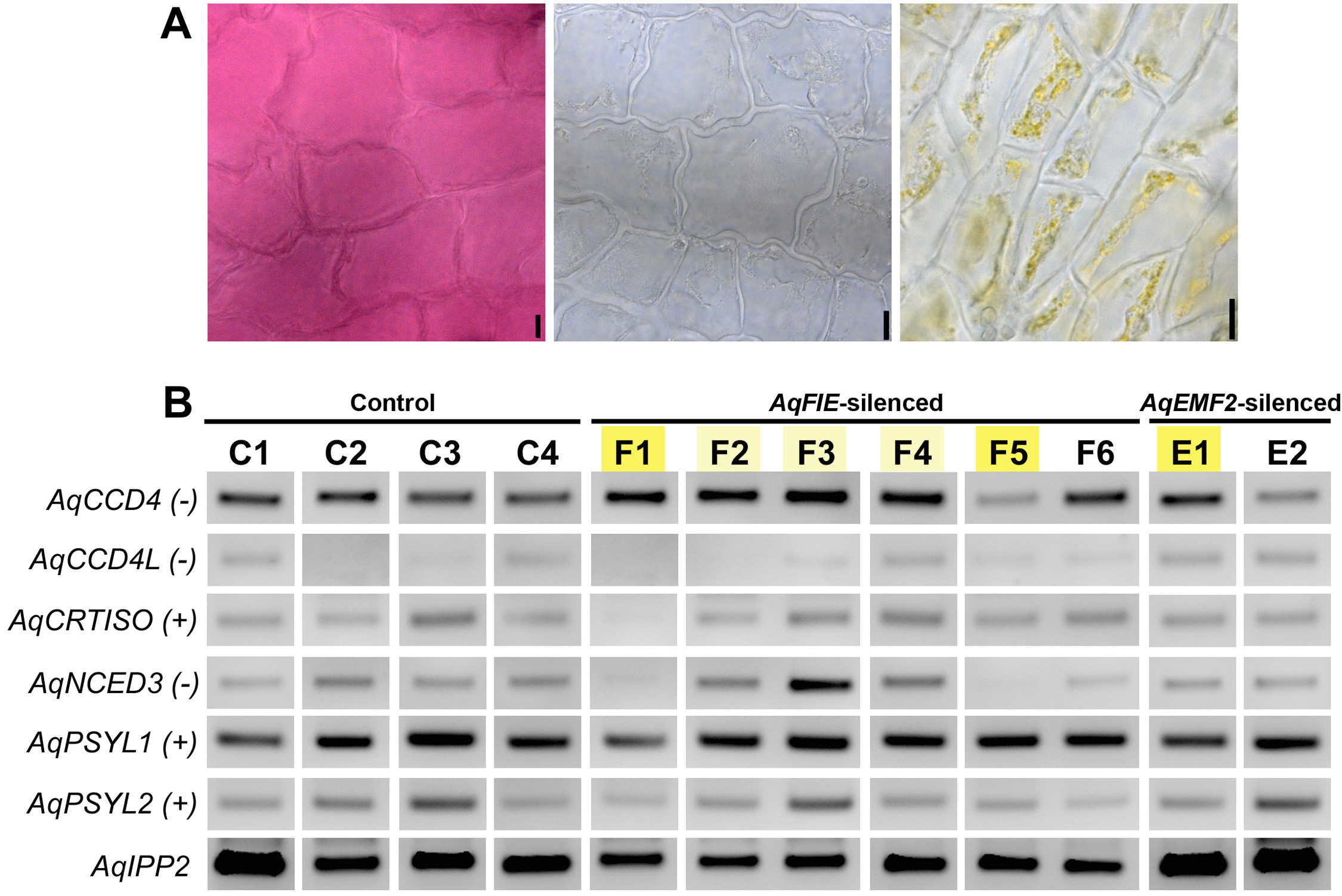

Supplement: Additional file 3 — The PRC2 regulates carotenoid production in A. coerulea petals. A. High magnification views of epidermal cells in A. x coerulea petal limbs. From left to right: Anthocyanin of untreated petal limb (anthocyanin is deposited in the vacuole, resulting in a very even distribution of color), almost complete lack of color in AqANS-silenced petal limb, and punctate pattern of carotenoid deposition in plastids of AqEMF2-silenced petal limb. B. Expression of several A. x coerulea homologs of genes important in carotenoid production (CRTISO and PSY) and degradation (CCD4 and NCED3) in AqANS-silenced control petals (C1-C4) and AqFIE (F1-F6) and AqEMF2 (E1 and E2) treated petals. Petals with strong yellow pigment are highlighted in dark yellow (F1, F5, and E1) and petals with pale yellow pigment are highlighted in light yellow (F2-F4). The expression of these genes is not consistently affected in the AqFIE and AqEMF2 silenced petal samples. It is possible that other genes in the carotenoid pathway are being misexpressed. Scale bars: 10 μm. [file 1471-2229-13-185-S3.jpeg]

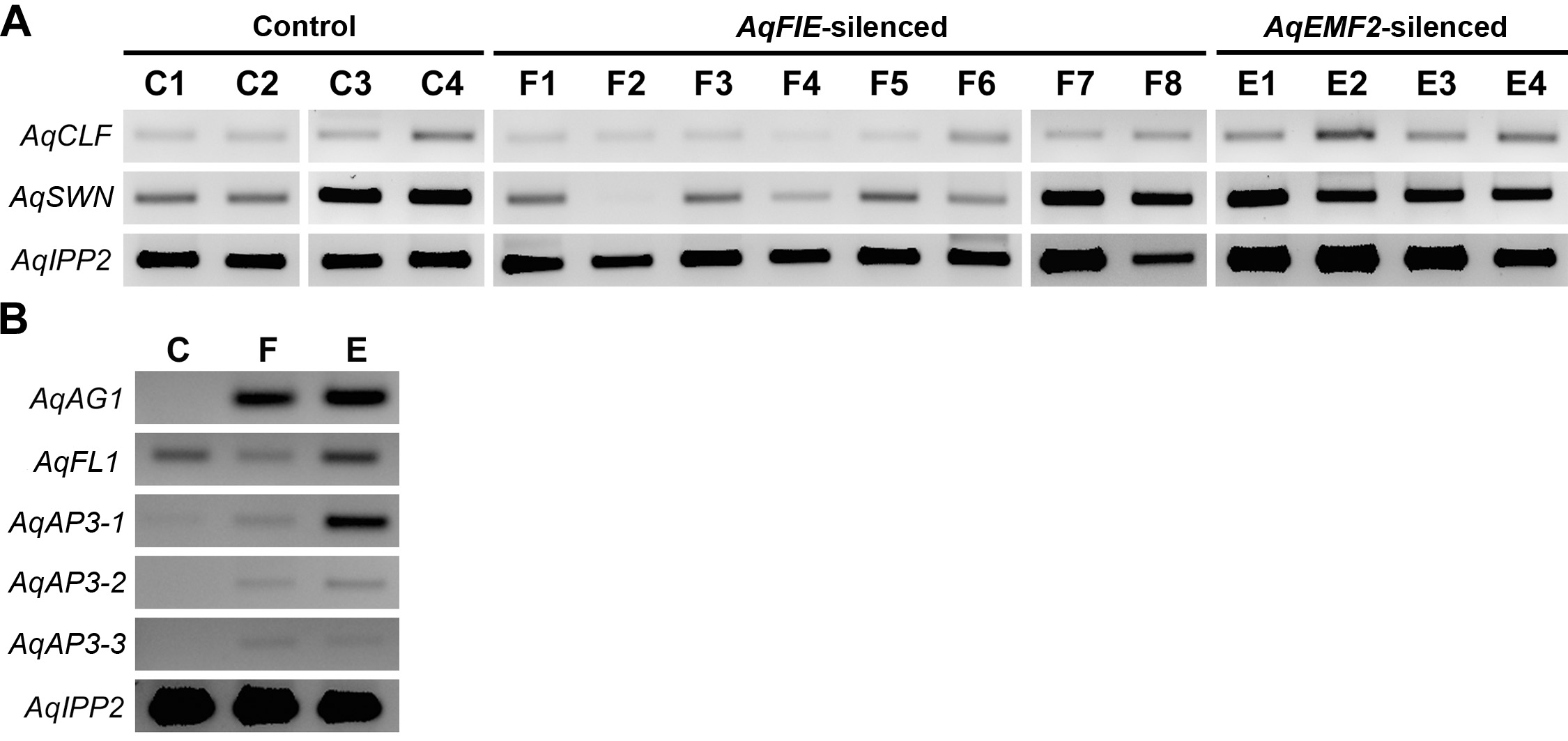

Supplement: Additional file 4 — Additional candidate gene expression in PRC2 VIGS-treated leaves. A. Expression of AqCLF and AqSWN in AqFIE- and AqEMF2-treated leaves. Although AqEMF2 appears to be down-regulated in some AqFIE-silenced leaves, the expression of AqCLF and AqSWN in these leaves is not affected. B. Expression of AqAG1, AqFL1, AqAP3-1, AqAP3-2, and AqAP3-3 in pooled AqANS silenced control leaves (C) and AqFIE (F) and AqEMF2 (E) silenced leaves. AqAP3-1 AqAP3-2 and AqAP3-3 is moderately up-regulated in both AqFIE and AqEMF2 silenced tissue while AgFL1 expression is unaffected. [file 1471-2229-13-185-S4.jpeg]
